# Beyond the T-Score: A Narrative Clinical Review of Adult Endocrine Osteoporosis Care

**DOI:** 10.3390/jcm15187258

**Published:** 2026-09-18

**Authors:** Laura Monica Georgescu, Andreea-Diana Igna, Emilia Cristina Popa Tăut, Bianca Anamaria Toderaș Herlaș, Ioana Adela Rațiu, Luis Miguel Luengo Perez, Nicoleta Ana Maria Pașcalău

**Affiliations:** 1 Faculty of Medicine and Pharmacy, University of Oradea, 1st December Square 10, 410073 Oradea, Romania; emilia.cristinapopa@gmail.com (E.C.P.T.); toderas.biancaanamaria@student.uoradea.ro (B.A.T.H.); ioana.ratiu@didactic.uoradea.ro (I.A.R.); nicoleta.pascalau@didactic.uoradea.ro (N.A.M.P.); 2 Department of Medical Sciences, Endocrinology Discipline, Faculty of Medicine and Pharmacy, University of Oradea, 1st December Square 10, 410073 Oradea, Romania; 3 Doctoral School of Biomedical Sciences, Faculty of Medicine and Pharmacy, University of Oradea, 410073 Oradea, Romania; 4 Nephrology Department, Bihor County Emergency Clinical Hospital, 12 Corneliu Coposu Street, 410469 Oradea, Romania; 5 Medical Sciences Department, Faculty of Medicine and Health Sciences, University of Extremadura, 06006 Badajoz, Spain; luismluengo@unex.es; 6 Clinical Nutrition and Dietetics Unit, Endocrinology and Nutrition Section, Badajoz University Hospital, 06008 Badajoz, Spain; 7 Department of Psycho Neuroscience and Recovery, Faculty of Medicine and Pharmacy, University of Oradea, 410087 Oradea, Romania

**Keywords:** osteoporosis, fragility fracture, fracture risk, T-score, trabecular bone score, denosumab, sequential therapy, anabolic therapy, fracture liaison service, endocrinology

## Abstract

**Background/Objectives:** Bone mineral density (BMD) is fundamental to osteoporosis assessment, but fracture risk is also shaped by fracture history, falls, frailty, secondary causes, age, sex, and treatment context. This narrative review examines clinically relevant information that modifies adult endocrine decision-making beyond the T-score. **Methods:** PubMed/MEDLINE was searched through 28 June 2026; recent guidelines, consensus statements, systematic reviews, meta-analyses, and pivotal fracture-endpoint trials were prioritized. The exact search strings, filters, eligibility criteria, selection process, and prioritized guidance documents are provided in PubMed MEDLINE search strategies. **Results:** BMD should be interpreted with fracture history and clinical risk factors, while the applicability of T-scores differs by age and sex. FRAX, vertebral imaging, and trabecular bone score can refine assessment in selected patients. Very-high-risk patients may warrant early osteoanabolic or dual-action therapy followed by antiresorptive treatment, whereas antiresorptives remain appropriate for many high-risk patients. Denosumab requires planned continuity and a follow-on strategy; advanced chronic kidney disease requires particular attention to mineral metabolism and hypocalcemia risk. **Conclusions:** Contemporary osteoporosis care expands rather than replaces densitometry. Endocrine management should integrate valid DXA interpretation, fracture-risk urgency, reversible contributors, drug-specific safety, treatment feasibility, monitoring, and the next treatment phase.

## 1. Introduction and Literature Search Strategy

Osteoporosis is a common metabolic bone disease whose clinical importance is determined principally by fragility fractures rather than by a densitometric threshold alone. Hip and vertebral fractures are associated with pain, loss of mobility and independence, subsequent fracture risk, and excess mortality. Despite the availability of dual-energy X-ray absorptiometry (DXA), validated fracture-risk tools, and effective antifracture therapies, underdiagnosis and undertreatment remain common, including after an established fragility fracture [1,2,3].

The T-score continues to have a central role in osteoporosis diagnosis and fracture-risk assessment, but its diagnostic use is age- and sex-dependent. In postmenopausal women and men aged 50 years or older, T-scores are preferred for densitometric classification. In females before menopause and males younger than 50 years, Z-scores are preferred; in particular, osteoporosis in a man younger than 50 years should not be diagnosed from BMD alone [4]. The limitation of the T-score is therefore not that it lacks clinical value, but that even when it is the correct densitometric metric, the same value can carry different implications in patients with different ages, fracture histories, fall risk, glucocorticoid exposure, renal function, or comorbidities. A recent vertebral fracture, for example, changes the urgency of treatment in a way that cannot be conveyed by BMD alone. Current guidance therefore places densitometry within a broader assessment that includes fracture history, clinical risk factors, and, when appropriate, FRAX, vertebral imaging, and trabecular bone score (TBS) [1,2,4,5,6,7,8,9].

This perspective is particularly relevant to endocrinology. Skeletal fragility may coexist with primary hyperparathyroidism, thyroid excess or iatrogenic TSH suppression, glucocorticoid exposure, diabetes, hypogonadism, malabsorption, chronic kidney disease, and other disorders in which the mechanism of bone loss influences both the diagnostic work-up and the choice of therapy [1,2,5,6].

This article was prepared as a targeted narrative review rather than a systematic review. PubMed/MEDLINE was searched using combinations of terms grouped around five clinical themes: fracture-risk assessment; care delivery; pharmacologic treatment and sequencing; secondary osteoporosis; and special adult populations. The search was initially completed on 3 June 2026 and checked for relevant updates through 28 June 2026. English-language guidelines, consensus statements, systematic reviews, meta-analyses, and pivotal randomized or extension trials were prioritized, with preference for recent guidance and higher-level evidence when available. Reference lists of major guidelines and consensus documents were checked to recover landmark fracture-endpoint trials that remained clinically relevant. Adult human populations were eligible when the source informed diagnosis, risk stratification, endocrine evaluation, treatment selection, sequencing, safety, or monitoring. Pediatric osteoporosis, oncology-specific bone loss, transplant bone disease, rare monogenic skeletal disorders, conference abstracts without a full report, and sources without direct clinical relevance to the prespecified themes were excluded. Screening and selection were not performed independently in duplicate. Consequently, there were no paired independent screening disagreements; citations of uncertain relevance were discussed within the author team and retained when consensus supported inclusion, without formal third-reviewer adjudication or an inter-reviewer agreement statistic. No formal risk-of-bias assessment, GRADE certainty rating, or quantitative pooling was undertaken. For major therapeutic statements, fracture-endpoint randomized evidence was distinguished from BMD-endpoint transition studies, observational data, regulatory safety information, and expert consensus when this distinction affected interpretation. The exact reproducible PubMed strings, date and language limits, eligibility criteria, selection process, and prioritized guideline and consensus documents are reported in Supplementary Table S1. These methodological boundaries are considered explicitly in Section 7.3.

The added value of this review is not a new risk score or a competing guideline. It is a single clinically oriented pathway that connects technical DXA validity, fracture recency and multiplicity, FRAX and TBS refinement, endocrine and metabolic contributors, treatment intensity, drug-specific safety, therapeutic sequence, and long-term continuity. These elements are commonly distributed across separate densitometry positions, disease-specific guidelines, regulatory documents, and pharmacologic reviews. Bringing them together clarifies when information beyond BMD can change diagnosis, urgency, treatment selection, safety assessment, monitoring, or the next treatment phase in adult endocrine practice.

## 2. Beyond the T-Score: Care Gaps, Fracture Risk, FRAX, TBS, and Imminent Risk

### 2.1. From Treatment Gap to Care Gap

The traditional term treatment gap describes the proportion of eligible patients who do not receive osteoporosis medication, but it does not capture the full sequence of missed opportunities that can occur before and after a prescription. Patients may not be screened when screening is appropriate, a low-trauma fracture may be treated without triggering osteoporosis evaluation, vertebral fractures may remain unrecognized, or a patient may discontinue treatment without a safe transition plan. Recent literature has therefore proposed the broader concept of an osteoporosis care gap [3].

Fracture liaison services (FLS) address one of the most important components of this gap by systematically identifying patients after fragility fracture and linking the acute event to secondary fracture prevention. Meta-analytic evidence supports improved secondary-prevention processes and a reduction in subsequent fractures in many FLS models; reported mortality benefits are less certain because program structure varies and much of the mortality evidence is observational [10]. In settings without a formal FLS, the same principle can be applied through local pathways that assign responsibility for fracture-risk assessment, secondary-cause evaluation, treatment initiation, adherence, and follow-up.

### 2.2. Risk Stratification Beyond BMD

BMD is strongly associated with fracture risk, but fracture probability is influenced by more than bone mass. Age, prior fracture, parental hip fracture, glucocorticoid exposure, smoking, alcohol use, falls, frailty, and selected comorbidities alter the clinical meaning of a measured T-score [1,2,5,6]. Risk stratification is useful because it links the severity and immediacy of fracture risk to treatment intensity. Several contemporary frameworks distinguish high from very high fracture risk, particularly when fractures are recent, multiple, vertebral or hip in location, occur during adherent therapy, or coexist with very low BMD [6,8,11].

The distinction is clinically relevant rather than semantic. For many patients at high risk, an oral or intravenous bisphosphonate or another antiresorptive agent is an appropriate initial treatment. In patients with very high or imminent fracture risk, rapid and substantial fracture-risk reduction becomes a priority, and an osteoanabolic or dual-action strategy may be preferred when available and clinically suitable [6,8,11,12,13,14].

There is, however, no single universally accepted definition of very high fracture risk. NOGG, AACE/ACE, ESCEO/IOF, the Endocrine Society, and the ASBMR/BHOF goal-directed framework overlap in emphasizing recent or multiple fractures, very low BMD, fracture during therapy, and other markers of imminent risk, but the exact criteria and intervention thresholds differ [6,8,11,12,15,16]. Table 1 summarizes these frameworks without attempting to harmonize them. The categories and treatment-intensity examples used in this review are illustrative clinical syntheses, not new recommendations. Eligibility for a specific treatment must follow the guideline, product license, contraindications, and reimbursement rules applicable in the clinician’s jurisdiction.

### 2.3. FRAX and Its Limits

FRAX estimates 10-year probability of major osteoporotic fracture and hip fracture using age and selected clinical risk factors, with or without femoral-neck BMD. It is useful for translating multiple risk factors into an absolute probability and for supporting intervention decisions in health systems that use probability-based thresholds [5,6,11]. FRAX models and intervention thresholds are country- and healthcare-system-specific; a probability that triggers treatment in one setting may not do so in another. FRAX is a baseline risk-estimation tool rather than a validated instrument for monitoring treatment response, and its standard use is not a substitute for etiologic assessment in younger adults in whom conventional T-score-based algorithms may not apply. Falls are not directly incorporated, fracture recency and multiplicity are incompletely represented, and the effects of glucocorticoids and several comorbidities may require clinical adjustment. A recent major fragility fracture can therefore justify a more urgent treatment approach even when the 10-year estimate does not appear extreme [4,6,8,11,15].

### 2.4. Trabecular Bone Score and Vertebral Imaging

Before adding adjunctive measures, the DXA examination itself should be technically valid. Central DXA generally includes the PA lumbar spine and hip; the 33% (one-third) radius is particularly relevant when the hip or spine cannot be interpreted and in hyperparathyroidism [4]. Vertebrae affected by focal structural change or artifact should be excluded according to accepted densitometric rules, and diagnosis should not be based on a single remaining lumbar vertebra. Degenerative change, vertebral deformity, vascular calcification, positioning, and hardware can falsely alter lumbar-spine BMD. Longitudinal change should be interpreted against the facility-specific least significant change and, whenever possible, on the same or appropriately cross-calibrated device [4,17].

TBS is a texture-derived index calculated from lumbar-spine DXA images and provides information that is partly independent of areal BMD. The 2023 ISCD Official Positions state that TBS can refine fracture-risk stratification and can be used to adjust FRAX probability, particularly when a patient is close to a pharmacologic intervention threshold [9]. Its role should remain adjunctive: TBS should not be used as a stand-alone diagnosis of osteoporosis, as a substitute for hip and spine BMD, or as the sole reason to select a specific medication. TBS should be used only within the manufacturer’s recommended BMI range. L1–L4 may be retained in the presence of moderate degenerative change or chronic lumbar compression fracture, but TBS should not be reported when severe structural or pathological artifact invalidates interpretation, including vertebra plana, laminectomy, spinal hardware, or metastatic lesions [4,9]. Routine serial monitoring of TBS change is not currently recommended with the existing algorithm [4,9].

Vertebral fracture identification is equally important because a previously unrecognized vertebral fracture can materially reclassify risk. ISCD positions support vertebral fracture assessment (VFA) or lateral spine imaging in selected patients with low BMD and additional risk features, including advanced age, substantial historical height loss, an undocumented prior vertebral fracture, or sustained glucocorticoid exposure [4]. VFA is designed to detect vertebral fractures rather than to characterize every spinal abnormality; equivocal deformity, suspicious lytic or sclerotic change, or a finding that cannot be attributed confidently to osteoporosis should prompt conventional radiography or other clinically appropriate imaging [4].

### 2.5. Imminent Risk and Goal-Directed Treatment

Fracture risk is not constant over time. The period after a recent fragility fracture carries a particularly high probability of another fracture, which is why the term imminent fracture risk has become clinically useful [8,16]. The term does not have one universally fixed time window or threshold; in practice it generally refers to the early post-fracture period, with risk highest soon after the index fracture and remaining elevated over the following one to two years. The 2024 ASBMR/BHOF position statement on goal-directed osteoporosis treatment emphasizes rapid fracture-risk reduction for patients with recent fractures and other very-high-risk features and proposes individualized BMD goals as one component of long-term management [8]. This approach does not replace clinical judgment or national reimbursement criteria, but it reinforces the principle that the initial treatment should be chosen with the expected treatment trajectory in mind. The principal clinical modifiers and their management implications are summarized in Table 2.

## 3. Endocrine Evaluation and Secondary Causes of Skeletal Fragility

The first osteoporosis consultation should establish whether the patient has predominantly postmenopausal or age-related osteoporosis, or whether a secondary disorder is contributing materially to skeletal fragility. The probability of a secondary cause is not the same in every patient; age, sex, fracture pattern, severity of BMD loss, biochemical abnormalities, medication exposure, and associated symptoms should determine the breadth of investigation [1,2,5,6].

A practical baseline evaluation commonly includes serum calcium, creatinine and estimated glomerular filtration rate, alkaline phosphatase, 25-hydroxyvitamin D, complete blood count, and selected liver and thyroid tests. Serum phosphate, parathyroid hormone, serum protein electrophoresis, celiac testing, urinary calcium, cortisol testing, testosterone in men, and other investigations should be added when the history, examination, or initial laboratory results justify them [1,2,5,6]. The aim is not to create a universal laboratory panel but to avoid missing a treatable disorder.

### 3.1. Hyperparathyroidism and Calcium-Phosphate Disorders

Primary hyperparathyroidism is particularly relevant because it can present with low BMD or fragility fracture and changes both the interpretation and management of osteoporosis. The Fifth International Workshop provides criteria for evaluation and management, including the role of serum calcium, PTH, renal assessment, nephrolithiasis, and skeletal involvement [21]. In this setting, cortical bone assessment is clinically relevant, and ISCD guidance supports measuring the 33% radius in patients with hyperparathyroidism [4]. In patients with unexplained hypercalcemia or a compatible pattern of bone loss, osteoporosis treatment should not proceed without clarifying the parathyroid disorder.

### 3.2. Thyroid, Adrenal, Gonadal, and Gastrointestinal Contributors

Overt hyperthyroidism increases bone turnover and is an established secondary cause of bone loss, whereas physiologic levothyroxine replacement that maintains an appropriate TSH should not be conflated with intentional or inadvertent chronic TSH suppression. Prolonged iatrogenic TSH suppression may adversely affect BMD in selected patients, especially postmenopausal women. These effects are clinically relevant in differentiated thyroid cancer, where the intensity and duration of suppression should be balanced against oncologic benefit and skeletal risk [22]. Hypercortisolism and chronic glucocorticoid therapy can cause rapid deterioration in bone strength, whereas hypogonadism in men and premature estrogen deficiency in women are important causes of secondary osteoporosis. Malabsorption, celiac disease, bariatric surgery, chronic liver disease, and nutritional deficiencies should be considered when the clinical history suggests them [1,2,5,6,20,23].

### 3.3. Why Mechanism Matters for Treatment

Identifying a secondary cause can change the treatment sequence, not merely add another diagnosis. Persistent hypercalcemia, advanced CKD-mineral and bone disorder, active malabsorption, severe vitamin D deficiency, or uncontrolled endocrine disease can alter drug safety, expected response, and monitoring. Treatment of the secondary cause and antifracture therapy are often complementary rather than mutually exclusive; in a patient with high or very high fracture risk, necessary endocrine evaluation should not create avoidable delays in fracture prevention.

### 3.4. Falls, Exercise, Nutrition, and Modifiable Risk

Fracture prevention is not purely pharmacologic. Falls are a major proximal event for many non-vertebral fractures, and a history of falls should prompt review of balance, lower-limb strength, vision, footwear, orthostatic symptoms, sedating or hypotensive medication, and environmental hazards. Exercise recommendations should be individualized rather than restricted reflexively because of a low T-score. A multidisciplinary UK consensus supports progressive resistance and impact exercise for bone strength, targeted strength and balance training for people at risk of falls, and spinal extension/postural work for people with vertebral fracture, with modification of impact level in frail patients or those with multiple low-trauma fractures [18].

Adequate calcium intake, correction of vitamin D deficiency, sufficient dietary protein, smoking cessation, moderation of excess alcohol intake, and preservation of muscle mass are supportive components of care [5,6,15]. Calcium and vitamin D supplementation should not be presented as substitutes for antifracture medication in a high-risk patient; their role is to correct deficiency and support safe, effective pharmacologic treatment. The practical objective is to reduce skeletal fragility and fall exposure at the same time.

## 4. Pharmacological Treatment: Antiresorptive, Osteoanabolic, and Dual-Action Strategies

### 4.1. Bisphosphonates

Bisphosphonates remain a mainstay of osteoporosis treatment. Oral alendronate or risedronate is appropriate for many patients when adherence, gastrointestinal tolerance, correct administration, and renal function are compatible with the product label. Renal thresholds are agent-specific rather than a class-wide interchangeable rule: current US labeling does not recommend alendronate when creatinine clearance is <35 mL/min and does not recommend risedronate when creatinine clearance is <30 mL/min [24,25]. For osteoporosis-dose intravenous zoledronic acid, serum creatinine and Cockcroft-Gault creatinine clearance should be assessed before each dose; treatment is contraindicated when creatinine clearance is <35 mL/min or acute renal impairment is present, and infusion should be withheld until normovolemia is restored if dehydration is suspected [26]. Local product information should take precedence. Intravenous zoledronic acid is useful when oral adherence is doubtful, gastrointestinal contraindications are present, or an infrequent parenteral regimen is preferable [5,6,12]. Bisphosphonate retention in bone allows consideration of a treatment holiday in selected patients, but a holiday is a risk-based decision rather than an automatic endpoint. Major guidelines commonly recommend reassessment after approximately five years of oral bisphosphonate therapy or three years of annual zoledronic acid in patients whose fracture risk has become low enough to consider interruption; continued treatment or a different strategy is appropriate when risk remains high [5,6,12,15]. A bisphosphonate holiday should therefore be distinguished from permanent treatment cessation, and the patient requires continued clinical reassessment during the interval.

### 4.2. Denosumab

Denosumab is a potent antiresorptive therapy with a clinical profile that differs fundamentally from bisphosphonates. In the FREEDOM trial, denosumab reduced vertebral, hip, and nonvertebral fractures in postmenopausal women with osteoporosis [27]. Because its effect is reversible and it is not retained in bone, six-monthly administration must be reliable. Interruption or discontinuation can lead to rapid rebound in bone turnover, loss of BMD, and vertebral fractures, particularly after longer exposure and in patients with previous vertebral fractures [28,29]. The future transition strategy should therefore be discussed when denosumab is started rather than only when it is stopped. Renal clearance is not required, but advanced CKD is not risk-free: severe hypocalcemia risk rises as kidney function worsens, especially in dialysis and CKD-MBD [30,31]. For CKD G4-G5D, a practical prevention protocol is to determine whether denosumab is appropriate with a clinician experienced in CKD-MBD; assess calcium, intact PTH, 25-hydroxyvitamin D, and 1,25-dihydroxyvitamin D and consider bone-turnover status before treatment; correct pre-existing hypocalcemia and manage CKD-MBD; provide adequate calcium and activated vitamin D; review concomitant calcimimetics or other calcium-lowering therapies; and educate the patient about symptoms requiring urgent assessment. Current US prescribing information recommends serum-calcium monitoring weekly for the first month after each injection and monthly thereafter in advanced CKD, with prompt treatment of hypocalcemia [31]. Supplement type and dose, dialysis timing, and additional phosphate or magnesium monitoring should be individualized with nephrology.

### 4.3. Teriparatide and Abaloparatide

Osteoanabolic therapy is most relevant when fracture risk is very high and a rapid, substantial skeletal response is desired. In the VERO trial, teriparatide reduced new vertebral and clinical fractures more effectively than risedronate in postmenopausal women with severe osteoporosis [32]. Abaloparatide also reduced new vertebral fractures in the ACTIVE trial [33]. These agents should be viewed as part of a treatment sequence because the gains achieved during the anabolic phase require subsequent antiresorptive therapy to be maintained [8,13,14].

### 4.4. Romosozumab

Romosozumab inhibits sclerostin and has both bone-forming and antiresorptive effects. FRAME demonstrated substantial vertebral-fracture reduction during the first year of romosozumab followed by denosumab, whereas ARCH showed lower fracture incidence with romosozumab followed by alendronate than with alendronate alone in a higher-risk population [34,35]. A numerical imbalance in serious cardiovascular events was observed during the first year of ARCH but not as a consistent signal across the trial program; this supports a precautionary risk assessment but does not by itself establish a simple causal relationship. Cardiovascular assessment should explicitly include prior myocardial infarction and stroke and should consider other major cardiovascular risk factors. Regulatory wording varies by jurisdiction: US labeling advises against initiation after myocardial infarction or stroke within the preceding year, whereas the European product information lists any previous myocardial infarction or stroke as a contraindication [36,37]. Local labeling therefore takes precedence. A recent meta-analysis supports the skeletal efficacy of romosozumab-based sequential treatment [38]. Antiresorptive treatment after the romosozumab course is required to preserve skeletal gains [12,13,34,38].

### 4.5. Selecting Treatment Intensity

An osteoanabolic-first strategy should not be presented as the default treatment for every patient with osteoporosis. Antiresorptive therapy remains appropriate for a large proportion of patients at high fracture risk. Osteoanabolic or dual-action treatment is most compelling when risk is very high, especially with recent major fracture, multiple vertebral fractures, very low BMD, or fracture despite appropriate treatment [6,8,11,13]. These treatment-intensity examples are illustrative and do not create new eligibility criteria. Drug choice must conform to locally applicable guidance, licensed indications, contraindications, and reimbursement, while also considering cardiovascular history, renal function, access, cost, patient preference, and the feasibility of the next treatment phase.

### 4.6. Other Selected Pharmacologic Options

The therapeutic discussion should not imply that contemporary osteoporosis care consists only of bisphosphonates, denosumab, and bone-forming agents. Selective estrogen-receptor modulators such as raloxifene or bazedoxifene can be considered in selected postmenopausal women, particularly when vertebral-fracture prevention is the main objective and venous-thromboembolic risk is low. Menopausal hormone therapy may be appropriate for fracture prevention in carefully selected younger postmenopausal women who also have menopausal symptoms and no major contraindication, but it is not a general substitute for osteoporosis-specific therapy in older or very-high-risk patients [5,12,15]. Drug selection therefore depends on fracture pattern, age, comorbidity, reproductive status, contraindications, route preference, and the expected duration and sequence of treatment. A practical comparison of these strategies is provided in Table 3, and selected pivotal fracture-endpoint trials are summarized in Table 4.

## 5. Sequential Therapy and Treatment Continuity

Osteoporosis is a chronic disease, but individual drug courses are often time-limited or require reassessment. The clinically relevant question is therefore not only which treatment to start, but what treatment is expected to follow it. This is especially important after osteoanabolic therapy and during denosumab discontinuation [8,13,14,28,29,42].

### 5.1. Osteoanabolic-to-Antiresorptive Sequencing

The sequence from an osteoanabolic agent to an antiresorptive is well supported biologically and clinically. Bone formation and BMD gains achieved during the anabolic phase can be consolidated with an antiresorptive agent. By contrast, prior exposure to potent antiresorptive therapy can modify the response to a subsequently administered osteoanabolic drug. DATA-Switch demonstrated that transition from teriparatide to denosumab continued to increase BMD, whereas transition from denosumab to teriparatide was associated with transient or progressive bone loss at several skeletal sites [42]. These findings illustrate why the order of treatment matters.

### 5.2. Denosumab Exit Strategies

Denosumab should not be stopped or materially delayed without a documented follow-on plan. Before the final intended injection, record the date on which the next dose would be due, reassess fracture risk and renal function, confirm calcium-vitamin D adequacy, and determine whether oral bisphosphonate administration and adherence are realistic. The ECTS position statement uses approximately six months after the final injection—the time the next denosumab dose would otherwise be due—as a practical anchor for initiating a potent antiresorptive [39]. Oral alendronate may be used when gastrointestinal tolerance, adherence, and renal function permit; intravenous zoledronic acid is commonly selected when oral treatment is unsuitable or adherence is uncertain. The choice is individualized according to denosumab duration, prior bisphosphonate exposure, renal function, and fracture risk [29,39].

After the transition, evaluate adherence and new fracture symptoms and reassess BMD, preferably on the same DXA system. Serum CTX, and sometimes PINP, can be checked where assays and local protocols are available; persistent elevation may support additional or repeated antiresorptive treatment, but proposed thresholds and schedules are not fully harmonized [29,39,43]. One zoledronic acid infusion does not suppress rebound reliably in every patient, particularly after longer denosumab exposure. If bone-turnover markers are unavailable, specialist protocols may use a planned second assessment or dose, but evidence remains consensus- and surrogate-endpoint based. Severe renal impairment may preclude usual bisphosphonate strategies and requires specialist planning rather than an untreated gap. New back pain or height loss should prompt evaluation for vertebral fracture. A missed denosumab injection is not a benign drug holiday [28,29,39].

### 5.3. Treatment Failure, Fracture on Therapy, and Reassessment

A fracture during therapy does not automatically prove pharmacologic failure. The first step is to verify adherence, administration technique, treatment duration, interval from treatment initiation, DXA comparability, and the presence of a new secondary cause or increased fall risk. A single fracture can occur despite effective therapy because no available treatment abolishes risk. Recurrent fractures, a significant BMD decline beyond least significant change, or persistently unfavorable bone-turnover-marker response despite adequate exposure provide stronger reasons to question efficacy or adherence and to reconsider treatment intensity [5,6,8,15]. In a patient who remains at very high risk, escalation or a change in treatment sequence may be appropriate.

### 5.4. What the Sequencing Evidence Does Not Yet Establish

The rationale for sequencing is strong, but the evidence should not be overstated. Fracture-endpoint randomized trials directly support the antifracture efficacy of individual agents and, for FRAME and ARCH, specific romosozumab-first sequences [34,35]. Other transition evidence, including DATA-Switch, is based primarily on BMD and bone-turnover outcomes rather than fractures [42]. Post-denosumab strategies rely on smaller interventional studies, observational cohorts, surrogate outcomes, and expert consensus [29,39]. Direct head-to-head fracture data for every possible sequence are not available. Most pivotal trials enrolled postmenopausal women; evidence is substantially thinner in men, advanced CKD, younger adults, and pregnancy-associated osteoporosis [16,23,42]. Table 5 labels the main evidentiary basis for each commonly discussed sequence so that randomized fracture evidence is not conflated with BMD outcomes or consensus.

## 6. Special Populations and Longitudinal Monitoring

### 6.1. Glucocorticoid-Induced Osteoporosis

Glucocorticoid-associated fracture risk can rise early after treatment begins and is not explained completely by BMD. Daily and cumulative dose, treatment duration, age, prior fracture, and the underlying inflammatory disorder all influence risk. The 2022 American College of Rheumatology guideline recommends early fracture-risk assessment and treatment according to risk category rather than waiting for advanced bone loss [20]. FRAX can contribute to assessment in age-appropriate adults, but glucocorticoid dose and the rapid early change in fracture risk require clinical interpretation rather than uncritical reliance on the calculated probability.

### 6.2. Diabetes Mellitus

Type 2 diabetes is a classic example of the limitation of a purely densitometric approach. Patients may experience increased fracture risk despite BMD values that are normal or only modestly reduced. Altered bone material properties, accumulation of advanced glycation end products, impaired bone turnover, neuropathy, visual impairment, sarcopenia, and falls all contribute to the skeletal phenotype [19]. BMD remains useful, but the clinician should recognize that standard risk estimates may not capture every diabetes-related contributor. TBS can be considered as an adjunctive risk-refinement measure when technically valid, but it should not be used as a diabetes-specific diagnostic test or as the sole determinant of treatment [9,19].

### 6.3. Thyroid Disease

Overt hyperthyroidism accelerates bone turnover and is an established secondary cause of bone loss. In differentiated thyroid cancer, chronic TSH suppression may also affect BMD, with the clearest concern in postmenopausal women. A systematic review and meta-analysis reported adverse BMD effects in this group, while the evidence is less consistent in premenopausal women and men [22]. The intensity of TSH suppression should therefore be individualized according to oncologic risk while skeletal risk is assessed separately.

### 6.4. Chronic Kidney Disease

In CKD, osteoporosis and CKD-mineral and bone disorder can coexist. In CKD G1-G3 without major biochemical abnormalities, osteoporosis assessment often resembles that used in the general population, while the distinction becomes increasingly important in G4-G5D because PTH abnormalities, altered bone turnover, mineral disturbances, and renal osteodystrophy can change both diagnosis and treatment safety [44]. The European consensus statement for CKD G4-G5D recommends integrating BMD and fracture history with calcium, phosphate, PTH, alkaline phosphatase, vitamin D status, and the clinical trajectory of kidney disease [44]. Drug choice also becomes more complex: bisphosphonate use is limited by renal function and evidence gaps, while denosumab can produce clinically important hypocalcemia in advanced CKD, with highest risk in dialysis and CKD-MBD [30]. Collaboration with nephrology is appropriate when advanced CKD or biochemical evidence of CKD-MBD is present.

### 6.5. Osteoporosis in Men

Osteoporosis in men remains underrecognized. In men aged 50 years or older, T-scores are appropriate for densitometric classification, whereas Z-scores are preferred in younger men and BMD alone should not establish the diagnosis of osteoporosis before age 50 [4]. Secondary causes are common and include hypogonadism, glucocorticoid use, alcohol excess, chronic pulmonary disease, malabsorption, renal disease, and hematologic disorders. The 2024 evidence-based guideline for osteoporosis in men emphasizes active case finding, evaluation for secondary causes, and pharmacologic treatment according to fracture risk [23]. However, male osteoporosis trials are fewer and often use BMD rather than fracture incidence as the primary endpoint; the fracture-endpoint evidence base and direct evidence for treatment sequences are therefore smaller than in postmenopausal women. Applying anabolic-first, transition, or goal-directed strategies to men should be described as extrapolation when the specific sequence has not been tested for fracture outcomes in male populations.

### 6.6. Pregnancy- and Lactation-Associated Osteoporosis

Pregnancy- and lactation-associated osteoporosis (PLO) is rare but clinically important because it affects young women and often presents with acute back pain and vertebral fractures during late pregnancy or early postpartum. Physiologic maternal bone loss during lactation is usually reversible; PLO represents a much less common fragility-fracture phenotype in which genetic predisposition, low pre-pregnancy bone mass, low BMI, nutritional factors, and other secondary contributors may be relevant [45,46,47]. Evidence for treatment is derived mainly from observational series and case reports rather than randomized trials. Evaluation should exclude secondary causes, ensure adequate calcium and vitamin D status, consider the timing of weaning and future pregnancy plans, and weigh fracture burden against limited reproductive safety data. Long skeletal retention is an additional consideration when bisphosphonates are contemplated in women who may become pregnant again, whereas experience with osteoanabolic and other agents remains limited and off-label in many jurisdictions. No single pharmacologic sequence can currently be presented as an evidence-based standard for all women with PLO [46,47].

### 6.7. Monitoring Treatment Response

Monitoring should answer a clinical question rather than follow a fixed calendar. The 2023 ISCD Official Positions recommend that follow-up DXA be performed with a predefined objective and that the interval be individualized according to age, sex, fracture risk, treatment history, and the likelihood that the result will change management [17]. Comparisons should be made on the same device when possible and interpreted using the facility-specific least significant change; change should not be inferred from small numerical differences between non-cross-calibrated instruments [4,17]. New height loss, new back pain, a suspected interval vertebral fracture, or continued high vertebral risk may justify repeat VFA or conventional spine imaging rather than relying on BMD alone [4].

Bone turnover markers can provide earlier information than BMD about treatment effect and adherence. The recent ESCEO/IOF/IFCC consensus supports the use of standardized markers, particularly serum PINP for bone formation and beta-CTX for bone resorption, while emphasizing standardized sample collection and assay interpretation [43]. Markers are complementary to clinical follow-up and DXA; they do not replace fracture surveillance or assessment of adherence and secondary causes.

### 6.8. Long-Term Safety, Adherence, and Treatment Feasibility

Long-term planning also requires discussion of uncommon but clinically important adverse events. Osteonecrosis of the jaw and atypical femoral fracture are rare at osteoporosis treatment doses, but their possibility influences dental counseling, duration of potent antiresorptive therapy, and evaluation of persistent thigh or groin pain [5,6,15]. ISCD positions recommend attention to cortical abnormalities on femur DXA images and support bilateral full-length femur imaging in selected patients with prolonged bisphosphonate or denosumab exposure, particularly when clinical suspicion for atypical femoral fracture exists [4]. Concern about rare adverse events should be placed in the context of the substantially greater absolute fracture risk faced by appropriately selected high-risk patients; unsupervised treatment interruption can itself be harmful, particularly with denosumab.

Adherence and feasibility are equally important. A theoretically optimal sequence will fail if the patient cannot reliably follow oral dosing instructions, attend six-monthly injections, afford or access the next drug, or accept the proposed plan. Route of administration, renal function, comorbidity, cognitive and functional status, cost, local reimbursement, and patient preference should therefore be considered before treatment begins, not only after adherence problems emerge. The additional assessment priorities for selected populations are summarized in Table 6.

## 7. Practical Integration, Discussion, Limitations, and Conclusions

### 7.1. A Practical Endocrine Consultation

A useful osteoporosis consultation can be organized around a small number of decisions. First, confirm that the fracture history and DXA result are clinically and technically valid, including use of the correct T-score or Z-score framework for age and sex, appropriate skeletal sites, and recognition of artifact [4]. Second, establish prior fractures, including vertebral fractures that may not have been recognized clinically, and determine whether a recent fracture increases short-term risk. Third, assess falls, frailty, major clinical risk factors, and the need for FRAX or TBS refinement. Fourth, investigate secondary causes in a targeted manner and correct major nutritional or metabolic deficiencies. Fifth, classify current fracture risk using locally applicable guidance and select treatment intensity accordingly. Sixth, review drug-specific safety, renal function, cardiovascular history, reproductive context where relevant, route of administration, access, and patient preference. Seventh, address strength, balance, exercise, fall prevention, smoking/alcohol exposure, and adherence. Finally, define how efficacy and safety will be monitored and what therapy is expected to follow when the current course ends [4,5,6,8,9,11,15,16,17,18,19,20,22,23,30,39,43,44,45,46,47].

This approach avoids two opposite errors. One is undertreatment of a recently fractured or very-high-risk patient because the T-score does not appear sufficiently low. The other is overtreatment based on a densitometric threshold without considering competing risks, comorbidity, life expectancy, treatment feasibility, or patient preference. Risk stratification is therefore a method for improving treatment fit, not a reason to discard individualized judgment.

### 7.2. Discussion

**Figure 1:**
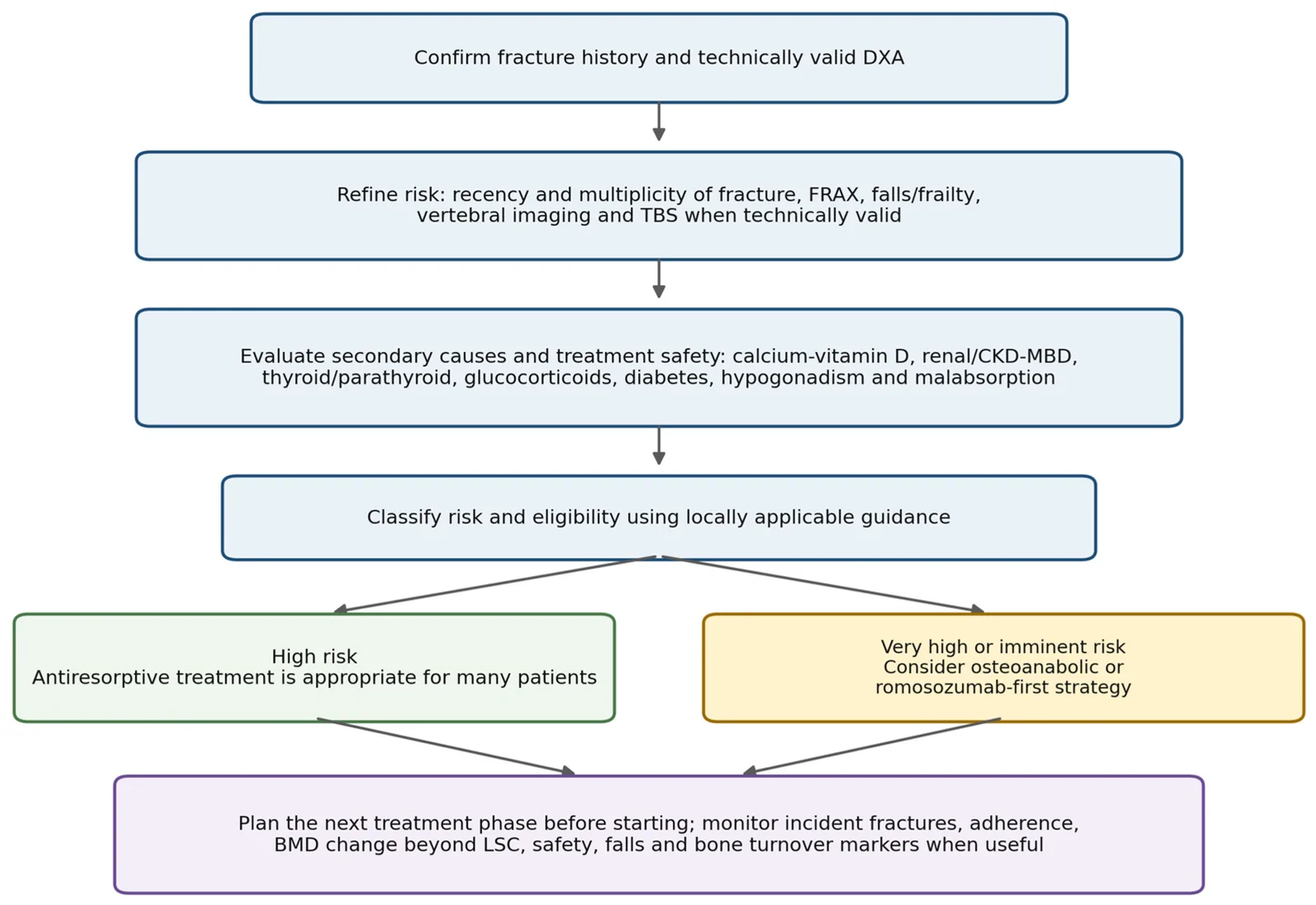
Proposed clinical pathway from DXA and fracture history to risk refinement, locally applicable treatment selection, sequencing, and monitoring. BMD, bone mineral density; CKD-MBD, chronic kidney disease-mineral and bone disorder; DXA, dual-energy X-ray absorptiometry; LSC, least significant change; TBS, trabecular bone score. Blue boxes represent sequential assessment steps; green denotes high risk; amber denotes very high or imminent risk; and purple denotes treatment continuity and follow-up.

Figure 1 condenses the review into a layered clinical pathway: establish a valid skeletal phenotype, refine the urgency of fracture risk, identify secondary causes and safety constraints, apply a locally recognized risk category, then select treatment only after planning its continuation. The certainty of evidence differs across these steps. Fracture-endpoint randomized trials anchor statements about drug efficacy; BMD and bone-turnover outcomes inform several transition strategies; observational evidence contributes to care-delivery and uncommon-population questions; and expert consensus remains necessary for denosumab exit protocols, selected very-high-risk definitions, and several special populations. These categories should not be treated as evidentially interchangeable.

The contribution of endocrinology is particularly relevant because osteoporosis often represents the final skeletal expression of several interacting mechanisms. A postmenopausal woman with TSH suppression, diabetes, CKD, or glucocorticoid exposure may have the same T-score as a patient with uncomplicated postmenopausal osteoporosis, yet the diagnostic priorities, safety considerations, and follow-up can differ substantially. The value of going beyond the T-score is therefore not the addition of more tests for every patient; it is the use of the right additional information when it can change management.

Goal-directed treatment provides a useful conceptual extension of this reasoning. The desired clinical outcome is fracture prevention, while BMD targets, absence of new fractures, adherence, and control of modifiable risk factors can help determine whether treatment is moving in the intended direction [8]. The evidence for specific targets is still developing and remains strongest in postmenopausal women, so the approach should not be applied rigidly to every population.

### 7.3. Limitations

This review is a targeted narrative synthesis and has several limitations. It was not preregistered, only PubMed/MEDLINE was searched directly, duplicate independent screening was not performed, and no formal risk-of-bias assessment, GRADE certainty rating, or quantitative pooling was undertaken; publication and selection bias are therefore possible. Borderline citations were resolved by author-team consensus rather than by formal third-reviewer adjudication. Reference-list checking was used to recover landmark fracture-endpoint trials, but the strategy was not intended to reproduce a systematic review. The English-language restriction may also have excluded relevant national guidance. The exact final-update search strings and selection framework are supplied to improve transparency and reproducibility, but they do not convert the work into a systematic review. The breadth of the topic required selective use of guidelines, consensus documents, major reviews, regulatory information, and pivotal trials rather than exhaustive coverage of every therapy, rare secondary cause, or national guideline. Definitions of high, very high, and imminent fracture risk are not harmonized, and FRAX calibration, intervention thresholds, drug licensing, contraindications, reimbursement, and access vary among healthcare systems. The diagnostic meaning of T-scores also differs by age and sex, so recommendations derived from postmenopausal populations cannot be transferred uncritically to younger adults. Evidence supporting drug sequencing is substantially stronger for postmenopausal women than for men, younger adults, pregnancy-related osteoporosis, and advanced CKD, and several transition studies use BMD rather than fracture outcomes as the principal endpoint. Denosumab exit strategies continue to evolve, and no single post-denosumab regimen is universally effective. Evidence for TBS as an adjunct to risk assessment is stronger than evidence for its serial use, while goal-directed BMD targets remain an evolving framework rather than a universally validated treat-to-target standard. Finally, this review addresses adult endocrine osteoporosis care and does not cover pediatric osteoporosis, oncology-specific bone loss, transplant bone disease, or rare monogenic skeletal disorders in depth. These limitations should constrain, rather than invalidate, clinical application of the synthesis.

### 7.4. Conclusions

Modern adult osteoporosis care requires both valid densitometry and clinical risk assessment. The T-score remains central in postmenopausal women and men aged 50 years or older, while younger adults require age-appropriate densitometric interpretation and greater emphasis on fracture context and secondary causes [4]. Across populations, fracture recency, multiplicity, falls, frailty, comorbidity, and treatment history can alter the urgency and feasibility of intervention. Antiresorptives remain appropriate for many high-risk patients; selected very-high-risk patients may benefit from an osteoanabolic or romosozumab-first strategy followed by antiresorptive therapy, but the evidence is strongest in postmenopausal women [6,8,13,15,16,27,32,33,34,35,38,40,41]. Treatment examples in this review are illustrative; local guidance, licensing, contraindications, and reimbursement determine eligibility. Treatment continuity should be planned from the outset, particularly with denosumab, and advanced CKD requires specific attention to CKD-MBD, mineral metabolism, and hypocalcemia prevention [28,29,30,31,39,44]. For endocrinologists, going beyond the T-score means using additional information only when it can improve diagnosis, safety, treatment intensity, or continuity of fracture prevention.

## Figures and Tables

**Table 1:** Comparison of high, very high, and imminent fracture-risk concepts across selected major frameworks.

Framework	High Risk	Very High or Imminent Risk	Important Limitation
AACE/ACE 2020 [15]	Osteoporosis or elevated fracture probability warranting pharmacologic treatment, without very-high-risk features.	Recent fracture (e.g., within 12 months), fracture on therapy or on skeletal-harm medication, multiple fractures, very low T-score (e.g., <−3.0), high fall risk, or very high FRAX probability (example thresholds: MOF > 30% or hip > 4.5%).	US-oriented examples; FRAX calibration, access, and product eligibility differ elsewhere.
NOGG 2024 [6]	FRAX probability at or above the age-dependent intervention threshold and below the very-high-risk threshold.	FRAX probability 60% above the intervention threshold; specialist-referral indicators include recent vertebral fracture within 2 years, at least 2 vertebral fractures, T-score ≤ −3.5, high-dose glucocorticoids, or multiple clinical risk factors, especially with a recent fracture.	Age-dependent UK thresholds and referral pathway should not be transferred numerically to other health systems.
ESCEO/IOF algorithm [11,16]	FRAX probability above the intervention threshold but below the upper/very-high-risk boundary.	Probability above the upper assessment/very-high-risk boundary, supporting consideration of an anabolic-first sequence when clinically appropriate.	Operational thresholds are country-specific and depend on locally selected FRAX intervention thresholds.
Endocrine Society 2020 [12]	Postmenopausal osteoporosis with prior hip/spine fracture, T-score ≤ −2.5, or FRAX probability meeting treatment thresholds.	Examples include severe osteoporosis (low T-score plus fracture) or multiple vertebral fractures; this category underpins selective use of romosozumab or PTH/PTHrP analogues.	Population and treatment recommendations are primarily for postmenopausal women.
ASBMR/BHOF 2024 [8]	Uses goal-directed reassessment rather than one universal high-risk cutoff.	Recent fracture and other features of imminent or very high risk favor rapid risk reduction and individualized treatment targets.	A position framework, not a replacement for local treatment or reimbursement criteria.

**Table 2:** Clinical features that modify fracture-risk interpretation beyond the T-score.

Clinical Feature	Why It Matters	Potential Management Consequence
Recent hip or vertebral fracture	Signals high short-term risk of another fracture [6,8]	Consider rapid treatment initiation and, when appropriate, a very-high-risk strategy.
Multiple vertebral fractures	Reflects substantial skeletal fragility [6,11]	May favor osteoanabolic or dual-action treatment followed by antiresorptive therapy.
Falls or frailty	Adds fracture risk not fully captured by BMD or FRAX [1,5,18]	Add fall assessment, strength/balance intervention, medication review, and environmental measures.
T2DM with relatively preserved BMD	Bone fragility may be underestimated by BMD alone [19]	Interpret DXA within the diabetic phenotype and other risk factors.
Low TBS	Adds information on trabecular texture and may adjust FRAX [9]	May refine risk when the treatment decision is borderline.
Unrecognized vertebral fracture	Can markedly reclassify risk [5,6,8]	Use vertebral imaging/VFA when clinically indicated.
Glucocorticoid exposure	Fracture risk depends on dose, duration, and clinical context [20]	Assess early; do not wait for severe BMD loss before prevention/treatment.

**Note:** “High” and “very high” fracture-risk categories are not defined identically across guidelines. The table summarizes recurring clinical features and should be interpreted alongside locally applicable thresholds and reimbursement criteria [6,8,11,15,16].

**Table 3:** Practical comparison of major pharmacologic strategies used in adult osteoporosis care.

Strategy	Typical Role	Important Advantages	Planning Issue/Limitation
Oral/IV bisphosphonates	Common first-line antiresorptive treatment in many high-risk patients [5,6,12,15]	Established fracture efficacy; residual skeletal effect permits risk-based interruption in selected patients	Renal function, oral administration requirements, and rare atypical femoral fracture or osteonecrosis-of-the-jaw risk with prolonged exposure; reassess before holiday
Denosumab	Potent antiresorptive option when continuous six-monthly treatment can be assured [5,12,39]	Strong antiresorptive efficacy; useful when oral bisphosphonates are unsuitable	No drug holiday; delayed or stopped treatment requires planned antiresorptive transition. Advanced CKD/CKD-MBD substantially increases hypocalcemia risk [30].
Teriparatide/abaloparatide	Selected very-high-risk patients, especially severe vertebral disease [8,16,32,33]	Rapid osteoanabolic effect; appropriate when a larger early skeletal response is needed	Time-limited course; must be followed by antiresorptive therapy; evidence strongest in postmenopausal women
Romosozumab	Selected very-high-risk patients [8,12,34,38]	Rapid BMD gains and fracture-risk reduction; dual action	Cardiovascular risk assessment and jurisdiction-specific contraindications; 12-month course followed by antiresorptive therapy
SERM/menopausal hormone therapy	Selected postmenopausal women [5,12,15]	Can address specific vertebral-fracture or menopausal-symptom contexts	Not appropriate for every fracture phenotype; thromboembolic, cardiovascular, breast/endometrial and age-related considerations vary by agent

**Table 4:** Selected pivotal fracture-endpoint trials that anchor the pharmacologic evidence base.

Trial/Treatment	Population	Comparator	Fracture Message	Interpretive Caveat
FIT/alendronate [40]	Postmenopausal women with prevalent vertebral fracture	Placebo	Reduced vertebral and clinical fracture risk	Older placebo-controlled population; does not define current treatment sequencing.
HORIZON-PFT/zoledronic acid [41]	Postmenopausal women with osteoporosis	Placebo	Reduced vertebral, hip, and other clinical fractures over 3 years	Infusion safety, renal function, and treatment duration require separate clinical assessment.
FREEDOM/denosumab [27]	Postmenopausal women with osteoporosis	Placebo	Reduced vertebral, hip, and nonvertebral fractures	Trial efficacy does not remove the need for a discontinuation plan or CKD-specific hypocalcemia assessment.
ACTIVE/abaloparatide [33]	Postmenopausal women with osteoporosis	Placebo (with teriparatide reference arm)	Reduced new vertebral fractures	Evidence base is primarily postmenopausal women; follow-on antiresorptive therapy is required.
VERO/teriparatide [32]	Postmenopausal women with severe osteoporosis	Risedronate	Lower new vertebral and clinical fracture incidence	Head-to-head evidence applies to the studied high-risk phenotype.
FRAME/romosozumab [35]	Postmenopausal women with osteoporosis	Placebo, then denosumab	Marked early vertebral-fracture reduction	Population and cardiovascular findings differ from ARCH; cross-trial comparisons are inappropriate.
ARCH/romosozumab [34]	Postmenopausal women at high fracture risk	Alendronate, then both groups alendronate	Lower vertebral, clinical, and hip fracture incidence with romosozumab-first sequence	Active-comparator design and cardiovascular event imbalance require context-specific interpretation.

**Note:** These trials enrolled different populations, used different comparators and follow-up periods, and should not be used for informal cross-trial ranking of drug efficacy. The table is intended to show the fracture-endpoint evidence underlying statements in the text.

**Table 5:** Common treatment sequences and points that require active planning. The arrow symbol indicates the intended treatment transition or sequence.

Sequence	Typical Clinical Context	Key Point	Principal Evidence Basis
Bisphosphonate → reassessment	Many high-risk patients [5,6,12]	Reassess fracture risk and BMD before considering a holiday.	Fracture-endpoint RCTs for bisphosphonate efficacy; holiday timing mainly guideline/extension evidence.
Teriparatide/abaloparatide → antiresorptive	Very high fracture risk [8,13,32,33]	Consolidation is required to maintain gains.	Initial-agent fracture RCTs; consolidation supported by BMD/transition data and guidelines.
Romosozumab → antiresorptive	Selected very-high-risk patients [34,38]	Assess cardiovascular risk before romosozumab and consolidate afterward.	Fracture-endpoint sequential RCT evidence from FRAME/ARCH, plus guideline recommendations.
Denosumab → planned bisphosphonate/antiresorptive	Whenever denosumab is discontinued [28,29,39]	Avoid unplanned gaps; plan alternative antiresorptive therapy around the time the next injection is due and monitor the transition.	Surrogate-endpoint interventional studies, observational data, and expert consensus; no universal fracture-validated exit schedule.
Fracture during therapy → reassessment	Incident fracture despite reported treatment [5,6,8]	Check adherence, DXA quality, secondary causes, falls, and need for escalation.	Guideline and expert-consensus reassessment framework; an isolated fracture is not automatically treatment failure.

**Table 6:** Selected special populations: what changes in the endocrine assessment.

Population	Additional Clinical Issue	Relevant Evidence
Glucocorticoid exposure	Risk rises early; dose and duration matter beyond BMD	ACR guideline [20]
Type 2 diabetes	Fracture risk may be high despite relatively preserved BMD	Lancet Diabetes Endocrinol review [19]
TSH suppression/thyroid disease	Skeletal risk is particularly relevant in postmenopausal women	Systematic review/meta-analysis [22]
CKD G4-G5D	Differentiate osteoporosis from CKD-MBD and assess mineral metabolism	European consensus [44]
Men	Search actively for secondary causes; treat according to fracture risk	Evidence-based guideline [23]
Pregnancy/lactation	Young age, vertebral presentation, fertility and reproductive safety considerations	Recent endocrine reviews; evidence remains observational [45,46,47]

## Data Availability

No new data were created or analyzed in this study. Data sharing is not applicable to this article.

## Supplementary Materials

The following supporting information can be downloaded at: https://www.mdpi.com/article/10.3390/jcm15187258/s1, Table S1: Reproducible PubMed/MEDLINE search strategies, eligibility criteria, selection process, and prioritized guideline and consensus documents.
