# Supplementary material for: Beyond the T-Score: A Narrative Clinical Review of Adult Endocrine Osteoporosis Care
Source: J Clin Med. 2026 Sep 18;15(18):7258. doi: 10.3390/jcm15187258 (PMC13607700; doi:10.3390/jcm15187258)
Supplement: Supplementary file 1 [file jcm-15-07258-s001.zip › jcm-4566070-supplementary.pdf]

Supplementary File S1

Beyond the T-Score: A Narrative Clinical Review of Adult Endocrine Osteoporosis Care

This file reports the search strategy used for the narrative review. It includes the full PubMed/MEDLINE search syntax, eligibility criteria, selection procedure, and the main guidelines and consensus documents considered.

Table S1 PubMed MEDLINE search strategies

Theme	Exact PubMed string	Use in the review
1. Risk assessment, DXA, FRAX, TBS, imminent risk	(("Osteoporosis"[Mesh] OR osteoporosis[Title/Abstract] OR "fragility fracture"[Title/Abstract] OR "fragility fractures"[Title/Abstract]) AND ("Fracture Risk Assessment"[Mesh] OR "fracture risk"[Title/Abstract] OR FRAX[Title/Abstract] OR "trabecular bone score"[Title/Abstract] OR TBS[Title/Abstract] OR "imminent fracture risk"[Title/Abstract] OR "very high fracture risk"[Title/Abstract] OR "goal-directed treatment"[Title/Abstract] OR "dual-energy x-ray absorptiometry"[Title/Abstract] OR DXA[Title/Abstract])) AND english[Language] AND ("2019/01/01"[Date - Publication] : "2026/06/28"[Date - Publication])	Assessment of fracture risk and interpretation of DXA, FRAX, and TBS.
2. Care gap and fracture liaison services	((osteoporosis[Title/Abstract] OR "fragility fracture"[Title/Abstract]) AND ("care gap"[Title/Abstract] OR "treatment gap"[Title/Abstract] OR "fracture liaison service"[Title/Abstract] OR "fracture liaison services"[Title/Abstract] OR "secondary fracture prevention"[Title/Abstract])) AND english[Language] AND ("2019/01/01"[Date - Publication] : "2026/06/28"[Date - Publication])	Organization of secondary fracture prevention services.
3. Pharmacologic treatment and sequencing	((osteoporosis[Title/Abstract] OR "fragility fracture"[Title/Abstract]) AND (bisphosphonate*[Title/Abstract] OR alendronate[Title/Abstract] OR risedronate[Title/Abstract] OR "zoledronic acid"[Title/Abstract] OR denosumab[Title/Abstract] OR romosozumab[Title/Abstract] OR teriparatide[Title/Abstract] OR abaloparatide[Title/Abstract]) AND (fracture*[Title/Abstract] OR "bone mineral density"[Title/Abstract] OR BMD[Title/Abstract] OR sequential[Title/Abstract] OR sequencing[Title/Abstract] OR transition*[Title/Abstract] OR discontinu*[Title/Abstract] OR "anabolic first"[Title/Abstract])) AND english[Language] AND ("2019/01/01"[Date - Publication] : "2026/06/28"[Date - Publication])	Drug efficacy, treatment sequences, discontinuation, and safety.
4. Secondary endocrine and metabolic osteoporosis	((osteoporosis[Title/Abstract] OR "skeletal fragility"[Title/Abstract] OR "fragility fracture"[Title/Abstract]) AND ("Glucocorticoid-Induced Osteoporosis"[Mesh] OR glucocorticoid*[Title/Abstract] OR hyperthyroid*[Title/Abstract] OR "TSH suppression"[Title/Abstract] OR hyperparathyroid*[Title/Abstract] OR diabetes[Title/Abstract] OR "chronic kidney disease"[Title/Abstract] OR CKD[Title/Abstract] OR hypogonadism[Title/Abstract] OR malabsorption[Title/Abstract])) AND english[Language] AND ("2019/01/01"[Date - Publication] : "2026/06/28"[Date - Publication])	Secondary causes relevant to investigation or treatment.
5. Special adult populations and monitoring	((osteoporosis[Title/Abstract] OR "bone fragility"[Title/Abstract]) AND (men[Title/Abstract] OR male[Title/Abstract] OR pregnancy[Title/Abstract] OR lactation[Title/Abstract] OR "pregnancy- and lactation-associated osteoporosis"[Title/Abstract] OR PLO[Title/Abstract] OR monitoring[Title/Abstract] OR "bone turnover marker"[Title/Abstract] OR PINP[Title/Abstract] OR CTX[Title/Abstract])) AND english[Language] AND ("2019/01/01"[Date - Publication] : "2026/06/28"[Date - Publication])	Evidence in men, pregnancy- and lactation-associated osteoporosis, and monitoring.
6. Guidelines, consensus, and position statements	((osteoporosis[Title/Abstract] OR "fragility fracture"[Title/Abstract]) AND (guideline[Publication Type] OR guideline*[Title] OR consensus[Title/Abstract] OR "position statement"[Title/Abstract] OR "official positions"[Title/Abstract])) AND english[Language] AND ("2019/01/01"[Date - Publication] : "2026/06/28"[Date - Publication])	Recent guidance relevant to the clinical themes.
7. Landmark fracture and transition studies without recent-date restriction	((FIT[Title/Abstract] AND alendronate[Title/Abstract]) OR (HORIZON[Title/Abstract] AND "zoledronic acid"[Title/Abstract]) OR (FREEDOM[Title/Abstract] AND denosumab[Title/Abstract]) OR (ACTIVE[Title/Abstract] AND abaloparatide[Title/Abstract]) OR (VERO[Title/Abstract] AND teriparatide[Title/Abstract]) OR (FRAME[Title/Abstract] AND romosozumab[Title/Abstract]) OR (ARCH[Title/Abstract] AND romosozumab[Title/Abstract]) OR ("DATA-Switch"[Title/Abstract] OR "DATA Switch"[Title/Abstract])) AND english[Language]	Pivotal studies published before 2019 or not retrieved by the date-limited searches.

Search and selection

PubMed/MEDLINE was searched initially on 3 June 2026 and checked again on 28 June 2026. The thematic searches were limited to English-language publications dated from 1 January 2019 to 28 June 2026. No date restriction was applied to the search for named pivotal trials.

Reference lists of relevant guidelines, consensus statements, and systematic reviews were also examined to identify earlier landmark trials and regulatory information relevant to clinical practice.

Screening was performed by one reviewer and was not duplicated. Records of uncertain relevance were discussed within the author group before the final decision on inclusion. No inter-reviewer agreement statistic or formal adjudication procedure was used.

The evidence was synthesized narratively. The review had no registered protocol and did not include a PRISMA flow diagram, formal risk-of-bias assessment, GRADE evaluation by the authors, or quantitative pooling.

Eligibility criteria

Category	Specification
Sources included	Adult human studies and guidance relevant to osteoporosis or fragility fractures. Sources were retained when they informed DXA interpretation, fracture-risk assessment, investigation of secondary causes, drug efficacy, treatment sequence, safety, service delivery, or monitoring.
Sources given priority	Current guidelines and consensus statements, systematic reviews and meta-analyses, pivotal randomized trials with fracture outcomes, and informative extension or transition studies. Observational studies were used for service delivery, uncommon populations, and adverse effects. Official regulatory information was consulted for prescribing details.
Sources excluded	Pediatric osteoporosis, oncology-related bone loss, transplant bone disease, rare monogenic skeletal disorders outside the scope of the review, non-English publications, conference abstracts without a full report, and studies unrelated to the clinical questions addressed.
Presentation of evidence	The manuscript distinguishes fracture outcomes from changes in BMD or bone-turnover markers and identifies evidence derived from observational studies, regulatory documents, or expert consensus when this affects interpretation.

Guidelines and consensus documents considered

Document	Use in the review	Citation
NOGG 2024 UK guideline	Risk thresholds, indicators of very high or imminent risk, treatment, and follow-up	Manuscript reference 5
AACE/ACE 2020 guideline	Risk categories and initial treatment in postmenopausal women	Reference 30
Endocrine Society guideline update 2020	Drug treatment, romosozumab, and treatment sequence	Reference 9
ESCEO/IOF algorithm and sequential-treatment consensus	Definitions of high and very high risk and the place of anabolic treatment	References 8 and 31
ASBMR/BHOF position statement 2024	Treatment goals and reassessment during long-term care	Reference 28
ISCD 2023 Official Adult Positions	DXA interpretation, vertebral assessment, TBS, and monitoring	References 29 and 36
ECTS position statement on denosumab discontinuation	Timing and choice of treatment after denosumab	Reference 32
European CKD G4-G5D consensus	Assessment and treatment safety in CKD G4-G5D	Reference 23
ESCEO evidence-based guideline for osteoporosis in men	Diagnosis and treatment of osteoporosis in men	Reference 24
ACR glucocorticoid-induced osteoporosis guideline	Assessment and treatment of glucocorticoid-induced osteoporosis	Reference 20
ESCEO/IOF/IFCC bone-turnover-marker consensus	Use of PINP and CTX in monitoring	Reference 27
